# Correction: Laomeephol et al. Exploring the Gelation Mechanisms and Cytocompatibility of Gold (III)-Mediated Regenerated and Thiolated Silk Fibroin Hydrogels. *Biomolecules* 2020, *10*, 466

**DOI:** 10.3390/biom16070925

**Published:** 2026-06-23

**Authors:** Chavee Laomeephol, Helena Ferreira, Supansa Yodmuang, Rui L. Reis, Siriporn Damrongsakkul, Nuno M. Neves

**Affiliations:** 1 Biomedical Engineering Research Center, Faculty of Engineering, Chulalongkorn University, Bangkok 10330, Thailand; chavee.l@student.chula.ac.th (C.L.); Supansa.Y@chula.ac.th (S.Y.); 2 Biomaterial Engineering for Medical and Health Research Unit, Faculty of Engineering, Chulalongkorn University, Bangkok 10330, Thailand; 3 3B’s Research Group, I3Bs-Research Institute on Biomaterials, Biodegradables and Biomimetics, University of Minho, Headquarters of the European Institute of Excellence on Tissue Engineering and Regenerative Medicine, AvePark-Parque de Ciência e Tecnologia, Zona Industrial da Gandra, 4805-017 Barco, Guimarães, Portugal; helenaferreira@i3bs.uminho.pt (H.F.); rgreis@i3bs.uminho.pt (R.L.R.); 4 ICVS/3B’s-PT Government Associate Laboratory, 4806-909 Braga/Guimarães, Portugal; 5 Research Affairs, Faculty of Medicine, Chulalongkorn University, Bangkok 10330, Thailand; 6 The Discoveries Centre for Regenerative and Precision Medicine, Headquarters at University of Minho, Avepark, 4805-017 Barco, Guimarães, Portugal; 7 Department of Chemical Engineering, Faculty of Engineering, Chulalongkorn University, Bangkok 10330, Thailand

In the original publication [1], there were two errors in the assembly of Figure 2A, both of which occurred during figure panel preparation and were not detected prior to submission or publication.

First error: the image corresponding to the 0.5 mM Au condition at Day 3 was mistakenly duplicated and incorrectly assigned to the 1 mM Au condition at Day 1. In the corrected figure, this image was replaced with the correct image for the 1 mM Au sample at Day 1.

Second error: the images representing the “No Au” condition at both Day 1 and Day 3 were unintentionally duplicated. Upon investigation of the raw data, it was determined that the “No Au” Day 3 image used in the original figure was by mistake selected from the image set of the “No Au” Day 1 condition. Further examination revealed that the image corresponding to “No Au” Day 3 condition from the primary experiment was missing. Consequently, an image of the “No Au” Day 3 condition obtained from a later independent replicate test, performed in the same experiment plan, was now included. Given that this image accurately reflects the morphological characteristics of the testing condition and is consistent with the complementary data presented in the study, it has been used in the assembly of the corrected Figure 2A. The corrected version of Figure 2A is provided below:

**Figure 2:**
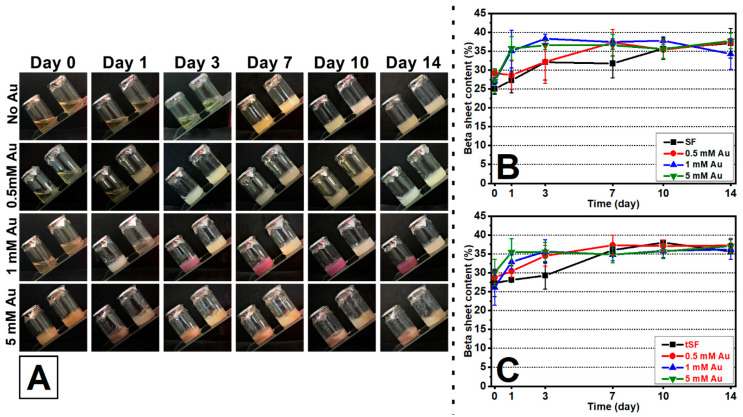
(**A**) Appearances of regenerated (no Au^3+^) and Au^3+^-mediated SF (left vial) and tSF (right vial) hydrogels incubated at 37 °C over 14 days. The amount of beta sheet determined from FTIR spectra using Fourier self-deconvolution (FSD) and curve-fitting techniques of the freeze-dried regenerated SF (**B**) and tSF (**C**) at different Au^3+^ concentrations.

The authors confirm that these corrections do not affect the data interpretation and scientific conclusions of the study. This correction was reviewed and approved by the Academic Editor. The original publication has been updated accordingly.
